# Real‐world clinical effectiveness and safety of CT‐P10 in patients with diffuse large B‐cell lymphoma: An observational study in Europe

**DOI:** 10.1002/jha2.593

**Published:** 2022-11-06

**Authors:** Mark J. Bishton, Gilles Salles, Camille Golfier, Wolfgang Knauf, Monica Bocchia, Deborah Turner, Borhane Slama, Jatinder Harchowal, Scott Marshall, Alberto Bosi, Juan José Bargay Lleonart, Manfred Welslau, SooKyoung Kim, Young N. Lee, Pier L. Zinzani, Kamel Laribi

**Affiliations:** ^1^ Nottingham City Hospital Nottingham University Hospitals NHS Trust Nottingham UK; ^2^ Translational Medical Sciences University of Nottingham Nottingham UK; ^3^ Centre Hospitalier Lyon Sud ‐ Service d'Hématologie Clinique Lyon France; ^4^ Centrum Für Hämatologie und Onkologie Bethanien Frankfurt am Main Germany; ^5^ Hematology Unit Azienda Ospedaliero Universitaria Senese, University of Siena Siena Italy; ^6^ Torbay and South Devon NHS Foundation Trust Devon UK; ^7^ Centre Hospitalier d'Avignon Avignon France; ^8^ University College London Hospitals NHS Foundation Trust London UK; ^9^ South Tyneside and Sunderland NHS Foundation Trust Sunderland Royal Hospital Sunderland UK; ^10^ Department of Clinical and Experimental Medicine University of Florence Florence Italy; ^11^ Son Llatzer University Hospital‐ Institut Investigació Sanitaria Illes Balear (IdIsBa) Palma de Mallorca Spain; ^12^ Onkologie Aschaffenburg, Klinikum Aschaffenburg Aschaffenburg Germany; ^13^ Celltrion Healthcare Co. Ltd. Incheon Republic of Korea; ^14^ IRCCS Azienda Ospedaliero‐Universitaria di Bologna Istituto di Ematologia “Seràgnoli” Bologna Italy; ^15^ Dipartimento di Medicina Specialistica e Sperimentale Università di Bologna Bologna Italy; ^16^ Department of Hematology Centre Hospitalier du Mans Le Mans France

**Keywords:** biosimilar, DLBCL, NHL, rituximab

## Abstract

The rituximab biosimilar CT‐P10 is approved for the treatment of non‐Hodgkin lymphoma. Previous studies have demonstrated clinical similarity between CT‐P10 and reference rituximab. However, real‐world data relating to treatment in patients with DLBCL with rituximab biosimilars are limited. This study collected real‐world data relating to the effectiveness and safety of CT‐P10 treatment from the medical records of 389 patients with DLBCL (24 centers, five European countries). For the primary outcome (clinical effectiveness), overall survival (OS), progression‐free survival (PFS), and best response (BR) were assessed. The percentage (95% confidence interval [95% CI]) of patients alive at 12‐, 18‐, and 30 months postindex (initiation of CT‐P10) was 86% (82.4%–89.4%), 81% (76.9%–84.9%), and 76% (71.2%–80.1%), respectively. The PFS rate (percent, [95% CI]) at 12‐, 18‐, and 30 months postindex was 78% (74.2%–82.5%), 72% (67.9%–76.9%), and 67% (61.9%–71.7%), respectively. Median OS/PFS was not reached. For 82% (*n* = 312) of patients, the BR to CT‐P10 was a complete response. Adverse events were consistent with known effects of chemotherapy. This international, multicenter study provides real‐world data on the safety and effectiveness profile of CT‐P10 for DLBCL treatment and supports the adoption of CT‐P10 for the treatment of DLBCL.

## 1 INTRODUCTION

CT‐P10 is a rituximab biosimilar approved for the treatment of rheumatic diseases and certain blood cancers, including non‐Hodgkin lymphoma (NHL) [1]. CT‐P10 is marketed as Truxima in the United States of America (USA) and Europe [2, 3] and is a rituximab biosimilar of Mabthera and Rituxan in Europe and the US, respectively [4, 5]. Since biosimilars often cost less than the reference drug, they have the potential to reduce the financial burden for healthcare systems and enable more patients access to rituximab treatment [6]. Indeed, since the emergence of approved biosimilars, real‐world evidence has demonstrated increasing biosimilar use across Europe [7].

Evidence of clinical similarity between CT‐P10 and reference rituximab has been described in pivotal studies of patients with rheumatoid arthritis [8, 9, 10, 11, 12, 13] and advanced follicular lymphoma (FL) [5, 14–16]. In the multinational phase III trial where patients with low tumor burden FL were treated with USA‐sourced rituximab or CT‐P10 monotherapy, CT‐P10 was well‐tolerated, and therapeutic equivalence to reference rituximab was demonstrated [16].

A major indication for rituximab is diffuse large B‐cell lymphoma (DLBCL), which is an aggressive NHL that accounts for approximately 30%–50% of all NHL cases [17]. The current standard of care for treatment of DLBCL is a regimen of cyclophosphamide, doxorubicin, vincristine, and prednisolone (CHOP) in combination with rituximab (R‐CHOP) [18].

Two real‐world studies of CT‐P10 in DLBCL patients have been published thus far. A single‐center real‐world study conducted in Korea investigated the safety and effectiveness of CT‐P10 in combination with CHOP (T[Truxima]‐CHOP) in patients with DLBCL and observed no significant difference in terms of response, overall survival (OS) or progression‐free survival (PFS) in patients treated with R‐CHOP compared to reference rituximab [19]. In addition, a recently published real‐world study from the Netherlands also demonstrated that OS was not significantly different in patients with DLBCL who received CT‐P10 or another rituximab biosimilar (GP‐2013) versus reference rituximab [20]. However, this study did not capture treatment‐related toxicities, including infusion‐related reactions (IRRs), or detailed treatment pattern data [20]. Collectively, the clinical trial and real‐world evidence data published to‐date demonstrate that CT‐P10 is associated with a similar clinical response, effectiveness, and safety profile to reference rituximab [14–16, 19].

To date, no multinational, multicenter studies have investigated the effectiveness or safety of CT‐P10 treatment in patients with DLBCL in a real‐world clinical setting. Additional real‐world data will help inform clinical management decisions and support the adoption of CT‐P10 for treatment of DLBCL in routine practice. The present study addressed this evidence gap by collecting real‐world data relating to the effectiveness and safety of CT‐P10 treatment in patients with DLBCL in several European countries.

## 2 METHODS

### 2.1 Study design and study setting

This study was a multicenter, noninterventional postauthorization safety study conducted in 24 European specialist treatment centers or hospitals (UK, 9; Spain, 1; France, 3; Germany, 7; Italy, 4), which routinely used CT‐P10 for DLBCL treatment. In the preindex observation period, patient‐level data for patients with DLBCL were collected from hospital medical records from the date of diagnosis until the index date (date of CT‐P10 initiation), to acquire demographic and clinical characteristics data. Safety and clinical outcome data were collected in the 30‐month postindex observation period or until patient death, if sooner.

### 2.2 Participants

Patients were eligible for inclusion if they had a confirmed diagnosis of DLBCL, received CT‐P10 for the treatment of DLBCL, were aged ≥18 years at date of DLBCL diagnosis, provided written informed consent for data collection (where required according to local regulations), and had preindex medical history available, with at least one clinical response assessment in the 30‐month postindex period (unless the patient was deceased). Patients receiving reference rituximab for previous treatment cycles (i.e., prior to the index event) within the same line of treatment were excluded. In addition, patients were excluded if their medical records were unavailable.

### 2.3 Patient consent and local approval

Approval was sought from institutional review boards and/or independent ethics committees and local hospitals, as appropriate for each country in the study. Further information can be found in the Supplementary Methods.

### 2.4 Endpoints and objectives

The primary objective was to describe the clinical effectiveness of CT‐P10 for the treatment of DLBCL. Endpoints addressing the primary objective were OS and PFS rate at 12‐, 18‐, and 30‐month postindex date, best response to CT‐P10 and time to complete or partial response. Secondary objectives included a description of baseline demographic, clinical and disease characteristics and assessment of the CT‐P10 safety profile and treatment patterns in the 30‐month postindex.

### 2.5 Statistical analyses

The initial target sample size for this study was 500 patients, to provide adequate precision for key descriptive outcomes in the overall sample and relevant subgroups. Demographic, clinical and disease characteristics data were described using summary statistics: distributions and descriptive statistics of both central tendency (medians and/or means) and dispersion (standard deviation [SD], interquartile range [IQR]) were presented for quantitative variables, as appropriate to the data distribution. For the primary outcome analysis (clinical effectiveness), time‐to‐event analyses were conducted using the Kaplan–Meier method, where OS and PFS were defined as the time from index until death (any cause), or the time from index until death from any cause or first documented evidence of disease progression, respectively. Patients were censored at 30‐month postindex date or the date of the last recorded hospital visit. Subgroup analysis for OS and PFS of patients who received CT‐P10 as the first line of therapy was also conducted. Disease progression and response data were assessed as documented in the patients’ medical records. Percentages were reported to the nearest whole number; hence, percentages may not total 100% due to rounding. Detailed information can be found in the Supplementary Methods.

## 3 RESULTS

### 3.1 Patient demographics and clinical characteristics

The study included 389 patients diagnosed with DLBCL with a median age of 69.7 (IQR 60.3–76.1) years at index (58%, [*n* = 227/389] male patients). The median duration of disease at index (days from diagnosis to index date) was 23 (IQR 12.0–46.0) days. The most common recorded comorbidities at index were diabetes (15%, [*n* = 57/389] of patients) and peripheral vascular disease (13%, [*n* = 51/389] of patients), and the most common Charlson Comorbidity Index score at index was “3” (23%, [*n* = 86/368]). Most patients had an Eastern Cooperative Oncology Group score at index of “0” (49%, [*n* = 116/237]) or “1” (30%, [*n* = 72/237]). Disease stage at index was recorded using the Ann Arbor or Lugano staging system, with 11% (*n* = 25/218) of patients recorded as stage I, 18% (*n* = 39/218) as stage II or stage III (*n* = 40/218), 52% (*n* = 113/218) as stage IV and <1% (*n* = 1/218) as other (unknown). Eighty‐four percent (*n* = 328/389) of patients received CT‐P10 as first‐line treatment. Most patients had an International Prognostic Index score at index of 2 (low‐intermediate, 29% [*n* = 76/263]) or 3 (high‐intermediate, 26% [*n* = 68/263]. Patient demographics and clinical characteristics are summarized in Tables 1 and 2, respectively.

**TABLE 1:** Patient demographics

Patient demographics	Overall (*n* = 389^1^)
Age at index date (years), median (IQR)	69.7 (60.3–76.1)
Male, *n* (%)	227 (58%)
Comorbidities at index, *n* (%)^2^	
Diabetes mellitus	57 (15%)
Peripheral vascular disease	51 (13%)
Malignancy (not related to DLBCL)	36 (9%)
Chronic pulmonary disease	29 (7%)
Rheumatologic disease	27 (7%)
Cerebrovascular disease	21 (5%)
Renal disease	20 (5%)
Congestive heart failure	15 (4%)
Myocardial infarction	13 (3%)
Liver disease	12 (3%)
Metastatic solid tumor	8 (2%)
Peptic ulcer disease	7 (2%)
HIV/AIDS	4 (1%)
Hemiplegia or paraplegia	1 (0%)
Dementia	0 (0%)
None recorded	188 (48%)
Charlson Comorbidity Index score at index date, *n* (% of 368)	
0	36 (10%)
1	34 (9%)
2	68 (18%)
3	86 (23%)
4	59 (16%)
5	42 (11%)
6	17 (5%)
7	8 (2%)
8	9 (2%)
≥9	9 (2%)
Not known	21

Abbreviations: DLBCL, diffuse large B‐cell lymphoma; IQR, interquartile range; HIV/AIDS, human immunodeficiency virus/acquired immunodeficiency syndrome.

^1^ Unless otherwise stated.

^2^ Data are not mutually exclusive.

**TABLE 2:** Clinical characteristics

Clinical characteristics	Overall (*n* = 389^1^)
Duration of disease at index (days from diagnosis to index date), median (IQR)	23 (12.0–46.0)
Distribution of disease duration (days), *n* (% of 389)	
Same day diagnosis	6 (2%)
1 < 10	64 (16%)
10 < 20	99 (25%)
20 < 30	59 (15%)
30 < 40	43 (11%)
40 < 50	31 (8%)
50 and over	87 (22%)
ECOG score at index^2^, *n* (% of 237)	
0	116 (49%)
1	72 (30%)
2	31 (13%)
3	12 (5%)
4	6 (3%)
Not recorded	152
DLBCL stage^3^ at index date, *n* (% of 218)	
I	25 (11%)
II	39 (18%)
III	40 (18%)
IV	113 (52%)
Other^4^	1 (<1%)
IPI score at index^5^, *n* (% of 263)	
0–1 (low)	56 (21%)
2 (low‐intermediate)	76 (29%)
3 (high‐intermediate)	68 (26%)
4–5 (high)	63 (24%)
Missing components	126
Position of CT‐P10 in the treatment pathway at index, *n* (%)	
First‐line	328 (84%)
Second‐line	41 (11%)
Third‐line	15 (4%)
Fourth‐line	2 (1%)
>Fourth‐line^6^	3 (1%)

Abbreviations: DLBCL, diffuse large B‐cell lymphoma; IQR, interquartile range; IPI, International Prognostic Index; ECOG, Eastern Cooperative Oncology Group.

^1^ Unless otherwise stated.

^2^ ECOG scores taken on the index date.

^3^ Stage represents both Ann Arbor and Lugano stages combined.

^4^ Other stages recorded = “stage 2 at least” (*n* = 1).

^5^ IPI score was recorded as documented, when available. For instance where the IPI score was calculated, DLBCL stage at index was used rather than stage at diagnosis.

^6^ Fifth‐line (*n* = 1); sixth‐line (*n* = 1), seventh‐line (*n* = 1).

### 3.2 Primary outcome: Clinical effectiveness of CT‐P10 treatment in patients with DLBCL

**FIGURE 1:**
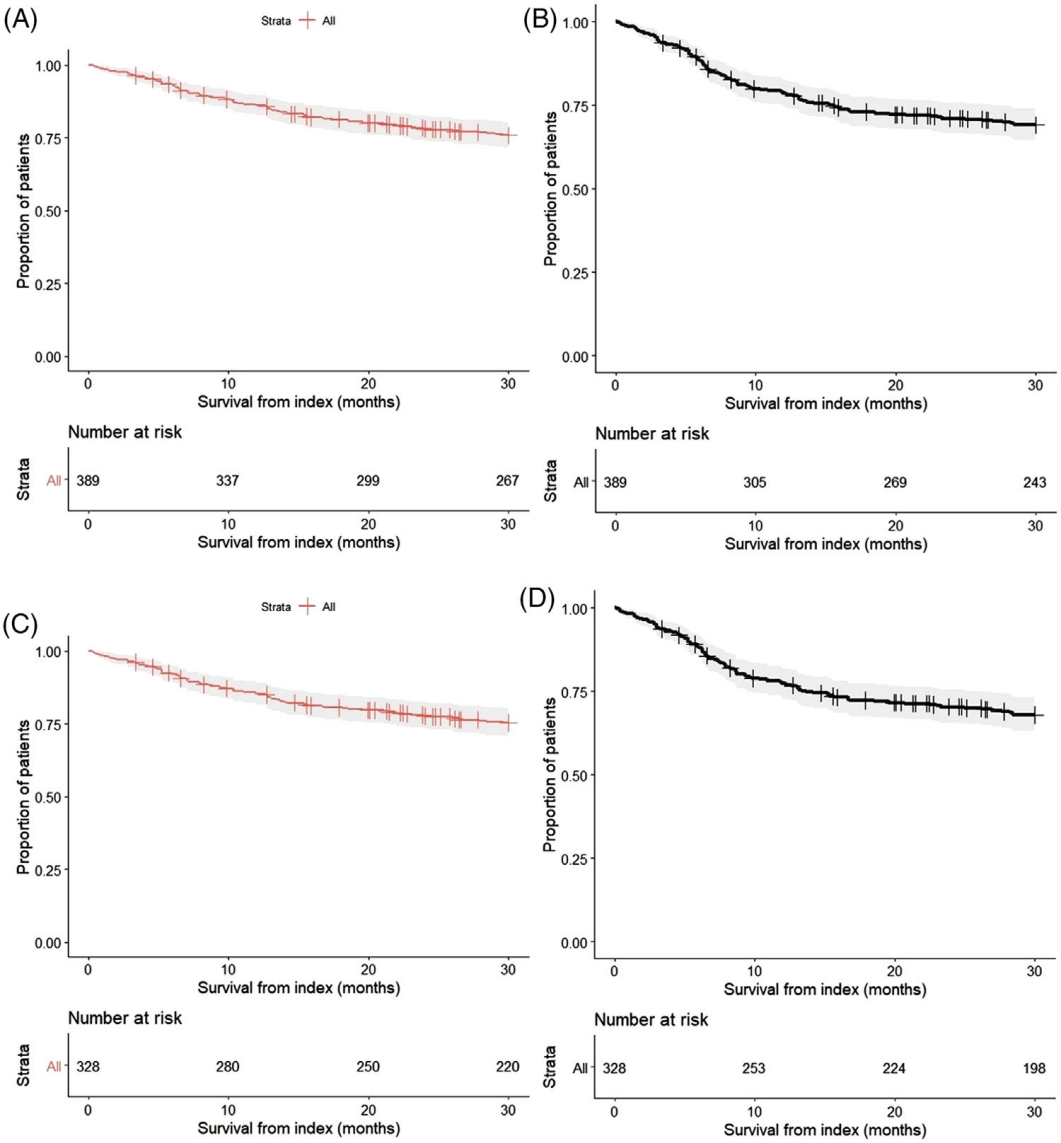
Kaplan–Meier charts for overall survival and progression‐free survival from index (months) for patients where CT‐P10 was the first line of treatment at index. Overall survival (OS) for all patients from index (A). Progression‐free survival (PFS) for all patients from index (B). OS for patients taking CT‐P10 as a first‐line of treatment (C). PFS for patients taking CT‐P10 as a first‐line of treatment (D). The assessment of disease progression was based on what was documented in patient medical records. This included the ‘Revized Response Criteria for Malignant Lymphoma’ [21] (if these criteria were used and documented locally).

**FIGURE 2:**
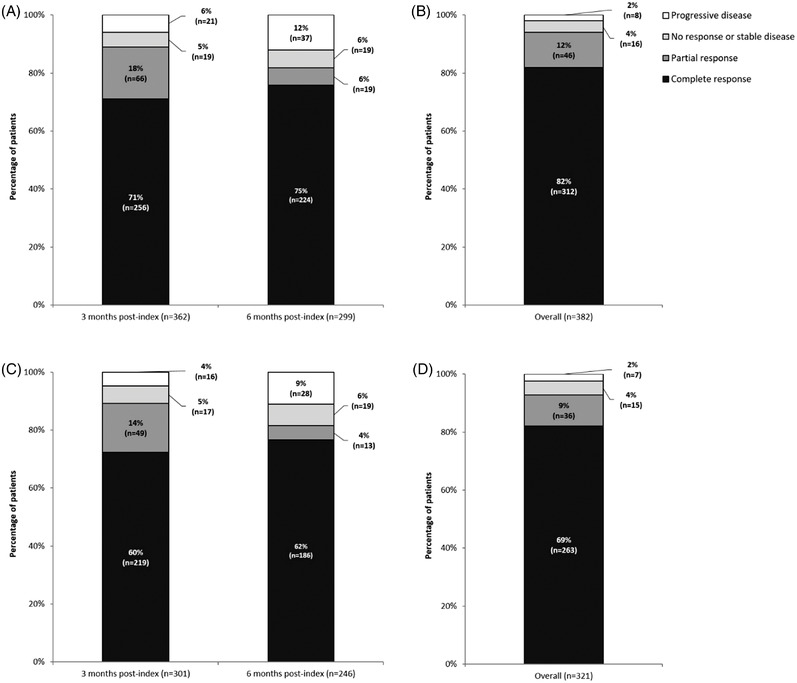
Overall and best response to CT‐P10. First response to CT‐P10 recorded on or after 3‐ and 6‐months postindex for all patients (A). Best response to CT‐P10 during the observation period for all patients (B). First response to CT‐P10 recorded on or after 3‐ and 6‐month postindex for patients receiving CT‐P10 as a first‐line treatment (C). Best response to CT‐P10 during the observation period for patients receiving CT‐P10 as a first‐line treatment (D)

The median (IQR) follow‐up was 30 (22.3–30.0) months. For the overall sample, the percentage (95% CI) of patients alive at 12‐, 18‐, and 30 months postindex was 86% (82.4%–89.4%), 81% (76.9%–84.9%), and 76% (71.2%–80.1%), respectively. The percentage (95% CI) of patients alive and free from disease progression at 12‐, 18‐, and 30 months was 78% (74.2%–82.5%), 72% (67.9%–76.9%), and 67% (61.9%–71.7%), respectively. For patients where CT‐P10 was the first line of therapy at index, the percentage (95% CI) of patients alive at 12‐, 18‐, and 30 months postindex was 86% (81.9%–89.5%), 81% (76.3%–85.0%), and 74% (69.2%–79.1%), respectively. Additionally, the percentage (95% CI) of patients alive and free from progression at 12, 18, and 30 months was 78% (73.8%–82.8%), 73% (67.9%–77.7%), and 67% (61.3%–72.1%), respectively; median OS/PFS was not reached (Figure 1). A total of 71% (*n* = 256/362) and 75% (*n* = 224/299) of patients achieved a complete response (CR) to CT‐P10 on or after 3‐ and 6 months, respectively. For patients where CT‐P10 was the first line of therapy, a total of 60% (*n* = 219/301) and 62% (*n* = 186/246) of patients achieved a CR to CT‐P10 on or after 3‐ and 6 months, respectively (Figure 1). The best recorded response to CT‐P10 was CR in 82% (*n* = 312/382) of patients, PR in 12% (*n* = 46/382), no response or stable disease in 4% (*n* = 16/382) and progressive disease in 2% (*n* = 8/382) Figure 2.

### 3.3 Treatment patterns

Patients received a median of 6.0 (range 1.0–18.0) CT‐P10 infusions during the observation period (including index and post‐index infusions), with almost two thirds of patients receiving 5<10 infusions (61% [*n* = 236/389]). R‐CHOP was the most frequently administered chemotherapy regimen at index (68%, [*n* = 266/389] of patients). At the end of the observation period 85% (*n* = 325/384) of patients had discontinued the first line of CT‐P10 treatment commenced at index due to planned completion of the treatment course, 5% (*n* = 18/384) due to adverse events (AEs), 5% (*n* = 21/384) due to disease progression and for 4% (*n* = 16/384) due to other reasons. For three patients (*n* = 3), the first line of treatment was ongoing at data collection. A complete response was the most common (*n* = 3/16) “other” reason for discontinuation of first‐line CT‐P10 treatment. Treatment pattern data are summarized in Table 3.

**TABLE 3:** Treatment patterns up to 30 months postindex in patients with DLBCL who received treatment with CT‐P10

Treatment information	Overall (*n* = 389^1^)
Chemotherapy regimen at first CT‐P10 infusion, *n* (%)	
R‐CHOP^2^	266 (68%)
R‐mini‐CHOP	28 (7%)
R‐CHOP with methotrexate	23 (6%)
R‐GCVP	9 (2%)
Rituximab monotherapy	8 (2%)
DA‐EPOCH‐R	4 (1%)
R‐CODOX‐M	1 (<1%)
Other (specify)^3^	50 (13%)
Reasons for discontinuation of first‐line CT‐P10 treatment, n (% of 384)	
Completed course of treatment	325 (85%)
Disease progression	21 (5%)
Adverse event	18 (5%)
Death	4 (1%)
Other^6^	16 (4%)
Not known	2
Not applicable (treatment ongoing at data collection)	3
Duration (days) of first‐line CT‐P10 treatment, median (IQR [*n* = 306])	113.0 (90.3–153.0)

*Note*: “R” in this table denotes biosimilar rituximab, CT‐P10.

Abbreviations: ACVBP, doxorubicin, cyclophosphamide, vindesine, bleomycin, and prednisone; CHOEP, cyclophosphamide, doxorubicin, vincristine, etoposide, prednisolone; CODOX‐M, cyclophosphamide and cytarabine (Ara‐C), vincristine (Oncovin), doxorubicin and methotrexate; DA‐EPOCH‐R, dose‐adjusted etoposide, prednisone, vincristine (Oncovin), cyclophosphamide, doxorubicin and rituximab; DECC, dexamethasone, etoposide, chlorambucil, lomustine; DHAC, dexamethasone, high dose cytarabine (Ara‐C) and carboplatin; DHAOx, dexamethasone, oxaliplatin and cytarabine (Ara‐C); EPOCH, etoposide, prednisone, vincristine (Oncovin), cyclophosphamide and doxorubicin; GEMOX, gemcitabine and oxaliplatin; IT, intrathecal; IQR, interquartile range; MATRix, methotrexate, cytarabine (Ara‐C), thiotepa and rituximab; MPV, methotrexate, procarbazine, vincristine; PMitCEBO, cyclophosphamide, mitoxantrone, etoposide, vincristine, bleomycin.

^1^ Unless otherwise stated

^2^ *n* = 3 regimens recorded as “CHOP” were recoded to R‐CHOP as CT‐P10 is the “R” (rituximab) in this regimen.

^3^ Other = R‐COMP (*n* = 9); R‐ACVBP (*n* = 4); R‐GDP (*n* = 4); R‐MPV (*n* = 4); R‐bendamustine (*n* = 3); R‐GEM‐P (*n* = 3); R‐mini‐COMP (*n* = 3); R‐GEMOX (*n* = 2); R‐DHAC (*n* = 2); R‐DHAOX (*n* = 2); TIER Trial: Thiotepa, etoposide, ifosfamide (*n* = 2); Ifosfamide + etoposide (*n* = 1); MATRix (*n* = 1); PMitCEBO+R (*n* = 1); R‐CCOP (*n* = 1); R‐CHOEP (*n* = 1); R‐CHOEP + IT methotrexate (*n* = 1); R‐DECC (*n* = 1); R‐EPOCH (*n* = 1); R‐ICE (*n* = 1); R‐MACOPB (*n* = 1); R‐mini CHOP‐cardioxane (*n* = 1); Rifampin (*n* = 1).

^4^ *n* = 13 regimens recorded as “CHOP” were recoded to R‐CHOP as CT‐P10 is the “R” (rituximab) in this regimen.

^5^ Other = R‐HOLOXAN‐VP16 (*n* = 21); R‐DHAOX (*n* = 18); R‐MPV (*n* = 15); R‐GEM‐P (*n* = 12); rituximab + bendamustine (*n* = 12); R‐ACVBP (*n* = 11); R‐GDP (*n* = 11); R‐bendamustine (*n* = 10); R‐CHOEP (*n* = 8); MATRix (*n* = 7); R‐mini‐COMP (*n* = 6); TIER Trial: thiotepa, etoposide, ifosfamide (*n* = 6); R‐CODOX‐M (*n* = 5); PMitCEBO+R (*n* = 5); R‐CEOP (*n* = 5); R‐COMP (50% dose) (*n* = 5); R‐ESHAP (*n* = 5); R‐CCOP (*n* = 4); R‐IVAC (*n* = 4); R‐methotrexate/cytarabine (Ara‐C) (*n* = 4); rituximab gemcitabine, oxaliplatin (*n* = 4); ifosfamide, etoposide (*n* = 3); R‐CHOEP and IT methotrexate (*n* = 3); R‐ICE‐methotrexate‐ibrutinib (*n* = 3); R‐MACOPB (*n* = 3); R‐DECC (*n* = 2); R‐EPOCH (*n* = 2); R‐GDC (*n* = 2); R‐GEMOX SD (*n* = 2); rituximab + bendamustine + polatuzumab vedotin (*n* = 2); R‐MPV + lenalidomide (*n* = 2); R‐ACVBP + methotrexate (*n* = 1); R‐cytarabine (Ara‐C) (*n* = 1); R‐DHAP (*n* = 1); rituximab + intrathecal methotrexate (*n* = 1); rituximab and temozolomide (*n* = 1); R‐mini CHOP‐cardioxane (*n* = 1); rifampin (*n* = 1); R‐MPV V2 (*n* = 1).

^6^ Other = complete response (*n* = 3); concern over increased risk of toxicity (*n* = 2); refractory disease, for palliation (*n* = 2); remission (*n* = 1); change of chemotherapy (*n* = 1); stable response (*n* = 1); no perceived benefit of continuing CT‐P10 treatment (*n* = 1); rituximab was administered subcutaneously (*n* = 1); medical decision side effect/comorbidity (*n* = 1); autologous transplantation (*n* = 1); lack of efficacy (*n* = 1); poor performance status (*n* = 1).

### 3.4 IRRs and AEs during the observation period

Overall, 90% (*n* = 351/389) of patients experienced one or more AEs (nonserious or serious, of any grade) at index or postindex, 83% (*n* = 324/389) experienced nonserious AEs and 43% (*n* = 168/387) experienced serious AEs (SAEs). Of all AEs with grade recorded (*n* = 2,337), most were classified as either grade 1 (49% [*n* = 1,153]) or grade 2 (31% [*n* = 715]). The percentage of AEs classified as grade 3, grade 4, and grade 5 was 16% (*n* = 369), 3% (*n* = 78), and 1% (*n* = 22), respectively. For all SAEs where the relationship to CT‐P10 was recorded (*n* = 193), SAEs were most commonly considered to be not related to CT‐P10 (64% [*n* = 123/193]). A total of 7% (*n* = 13/193), 3% (*n* = 5/193), 20% (*n* = 38/193), and 7% (*n* = 14/193) of SAEs were found to be definitely, probably, possibly or unlikely related to CT‐P10, respectively (the relationship to CT‐P10 was not recorded in most cases; 52% [*n* = 208/401]). IRR and AE data for the overall cohort and for first‐line CT‐P10 patients are summarized in supplementary Tables S1 and S2, respectively, and a summary of all AEs experienced at index or post index categorized by system organ class (SOC) can be seen in supplementary Table S3.

## 4 DISCUSSION

Given the benefits of biosimilar use for patients and potential cost‐savings for healthcare services, data relating to the safety and effectiveness of biosimilars such as CT‐P10 contribute to evidence‐based informed treatment decisions made by clinicians. Currently, published real‐world data pertaining to the safety and effectiveness of CT‐P10 for the treatment of DLBCL are limited. To‐date, patients with DLBCL have not been included in clinical trials of CT‐P10, and no studies have investigated the effectiveness or safety of CT‐P10 treatment in patients with DLBCL in a multinational, multicenter real‐world clinical setting. Hence, this study aimed to evaluate the real‐world clinical effectiveness and safety outcomes of CT‐P10 treatment in patients with DLBCL in Europe.

The primary outcomes of this study were related to the clinical effectiveness of CT‐P10 treatment in DLBCL patients when combined with standard of care chemotherapy; specifically, OS, PFS and treatment response were obtained.

While there are no clinical trial data relating to the use of CT‐P10 in patients with DLBCL, numerous studies have assessed the effectiveness and safety of reference rituximab [22, 23, 24, 25]. In addition, two real‐world studies describing the outcomes of CT‐P10 treatment in patients with DLBCL from Korea and the Netherlands have been published [19, 20].

In relation to clinical effectiveness, the results of the Korean real‐world study observed no significant differences in terms of CR, OS or PFS for CT‐P10 compared to reference rituximab. CR and 1‐year OS/PFS rates for CT‐P10 of 86.7% and 81.2% and 74.6%, respectively, were reported [19]. These data are broadly comparable to the CR rate of 82%, the 1‐year and 30‐month OS rates of 86% and 76%, respectively and the 1‐year and 30‐month PFS rates of, 78% and 67%, respectively, reported in the present study for the overall sample. In addition, the 3‐year OS rate of 73% in patients treated with rituximab biosimilars (R‐biosimilars [CT‐P10 and GP2014]) reported in the Netherlands real‐world study is comparable to the 30‐month OS rate in the present study [20].

A randomized trial was conducted in previously untreated elderly patients (aged 60–80 years) with DLBCL to compare CHOP chemotherapy in combination with rituximab versus CHOP chemotherapy alone. In relation to clinical effectiveness data, the 2‐year OS and PFS rates were 70% (95% CI 63–77 and 57% (95% CI [50–64]), respectively, in the group of patients who received CHOP chemotherapy in combination with rituximab. The percentage of patients achieving a complete and partial response was 52% and 7%, respectively [23].

A retrospective study by Sehn et al. (2005) investigated clinical outcomes in patients with newly diagnosed advanced‐stage DLBCL treated with CHOP plus rituximab [25]. The reported 2‐year OS and PFS rates were 73% and 68%, respectively. Taken together, the clinical effectiveness data reported in previous studies of reference rituximab [23, 25] and CT‐P10 [19, 20] Lee et al. (2020) studies relating to reference rituximab or CT‐P10 are broadly similar to the present study.

In some cases, as for OS/PFS and CR rate, our study appears to demonstrate a more favorable clinical effectiveness profile than patients who received reference rituximab in a previous study by Coiffier and authors [23], even with a longer observation period. Where, a 2‐year OS and PFS rate of 70% and 57% was reported, while, for our study, 30‐month OS and PFS rates were 76% and 67% respectively. The fact that patients in the above‐referenced study were exclusively elderly (60–80) may be a contributing factor; however, our median age was approximately 70 years.

In relation to the safety profile, in the present study, overall AE, SAE, and IRR rates of 90%, 43%, and 16%, respectively, were demonstrated. Select real‐world safety events relating to CT‐P10 treatment in patients with DLBCL were investigated by Lee et al. (2020). In this study, an overall IRR rate of 26.7% was presented for the CT‐P10 group, compared with 16% in the present study. Results from the present study from an SOC perspective indicate that AEs were primarily related to the gastrointestinal organ class, for example, nausea, vomiting, which are AEs consistent with the known effects of chemotherapy (Table S3) [19]. In this study, while data on the relatedness of SAEs were often missing, only a minority were considered to be related to CT‐P10.

Limitations are associated with this study. Due to the real‐world nature of this study, interpretation of data collected retrospectively was dependent on the completeness of the medical records and the reliability of the abstraction of data. For some participating countries, consent was a requirement and may have introduced selection bias (percentage of patients from each country). In addition, a total of 389 patients were included in this study as opposed to 500 patients that was initially planned, due to recruitment feasibility. However, as shown in Table S4, the upper and lower CIs for 300 patients were broadly similar to those calculated at 500. For example, for a survival rate of 80%, the CIs were 76.20 to 83.4 for 500 and 75 to 84.4 for 300 (which is smaller than our largest subgroup of 328). Our actual OS rate at 30 months was 76% (71.2%–80.1%), which is broadly in line with those estimates and supports our decision to terminate early, and therefore we are confident that the reduced sample size did not impact materially on the precision of OS and PFS estimates for the overall sample.

The originally planned number of 500 patients also enabled sufficient precision for subgroups as well as the overall sample, and only a single subgroup (*n* = 328) was reported.

An additional limitation is associated with the nonserious AE and SAE relatedness data. Collectively these data represent one of the main advantages of the present study; however, it is noted that in most cases relatedness was not available. Furthermore, in most cases, outcomes were assessed overall, irrespective of the line of CT‐P10 treatment. Hence, data for key clinical effectiveness and safety outcomes were stratified by first‐line CT‐P10 patients. Interestingly, OS, PFS (Figure 1), and key safety outcomes (Tables S1 and S2) were broadly similar for the overall cohort and for patients where CT‐P10 was the first line of therapy.

Despite these limitations, the described results will help to support treatment decisions and inform the routine clinical management of patients with DLBCL. Future research relating to prognostic factors and CT‐P10 for the treatment of DLBCL compared to rituximab is warranted and would further support treatment decisions. Despite these limitations, several strengths are associated with our study. For instance, data collected represent a multinational cohort from five European countries, which included CT‐P10‐related safety data such as IRRs. In addition, data are presented for the overall cohort, and for select endpoints (i.e., OS/PFS rates and response to CT‐P10), data were stratified by patients who received CT‐P10 as a first‐line treatment. Collectively, the results support the prescription of CT‐P10 for the treatment of DLBCL. Overall, it is evident that previously reported clinical effectiveness and safety data for reference rituximab are broadly similar to the data found in the present study in patients with DLBCL treated with CT‐P10.

## 5 CONCLUSION

In conclusion this is the first multinational real‐world study evaluating the clinical effectiveness and safety of CT‐P10 in patients with DLBCL. CT‐P10 was well tolerated in patients with DLBCL. The majority of AEs were mild or moderate and in line with the expected toxicity of chemotherapy. The CT‐P10 observed response, OS, and PFS rate in this study were similar to the rates found in other studies on reference rituximab and a single centre real‐world study on CT‐P10 treatment in DLBCL patients. Overall, the results from this study provide healthcare professionals with more detailed information on the safety profile CT‐P10 for the treatment of DLBCL, allowing for informed, evidence‐based decisions on the most appropriate treatment strategy for patients with DLBCL.

## AUTHOR CONTRIBUTIONS

SK, YNL, and PLZ designed the study. All authors performed the research, analyzed the data, and wrote the paper.

## CONFLICT OF INTEREST

MB has received research funding from Takeda, Gilead, Roche, Abbvie; Travel/acc/expenses = Roche, Takeda, Gilead, Celltrion, Honararia Tevapharma, Roche, Celltrion; Advisory board = Beigene; Trial Management Group = Roche. GS has received in the last 12 months financial compensations for participating in advisory boards or consulting from: Abbvie, Bayer, Beigene, BMS/Celgene, Epizyme, Genentech/Roche, Genmab, Incyte, Janssen, Kite/Gilead, Loxo, Milteniy, Morphosys, Novartis, Rapt, Regeneron and Takeda. Shareholder: Owkin. PLZ has received personal fees for consulting from Verastem, EUSA Pharma, MSD and Novartis; personal fees for participating in a speaker's bureau from Verastem, Celltrion, Gilead, Janssen‐Cilag, BMS, Servier, MSG, TG Therapeutics, Takeda, Roche, EUSA Pharma, Kyowa Kirin, Novartis, Incyte and Beigene; and personal fees for participating in advisory boards from Verastem, Celltrion, Gilead, Janssen‐Cilag, BMS, Servier, Sandoz, MSG, TG Therapeutics, Takeda, Roche, EUSA Pharma, Kyowa Kirin, Novartis, ADC Therapeutics, Incyte and Beigene. SM has received educational grants to attend educational meetings, educational lecturing and attendance of pharmaceutical advisory boards for Novartis, Abbvie and AstraZeneca in the last year. KL has received research grants from Celltrion. Outside this work, KL has received research grants from AbbVie, Novartis, Takeda, Roche, Amgen, and Sandoz as well as personal fees from AbbVie, Novartis, Sandoz, Celgene, Jansen, and Amgen. SKK and YNL are employees of Celltrion Healthcare. CG, WK, MBo, DT, BS, JH, AB, JJB, and MW have no conflict of interest to disclose.

## Supporting information

Supp Table information

Supplementary methods

## Data Availability

The data that support the findings of this study are available from the corresponding author upon reasonable request.
