# Supplementary material for: Real‐world clinical effectiveness and safety of CT‐P10 in patients with diffuse large B‐cell lymphoma: An observational study in Europe
Source: EJHaem. 2022 Nov 6;4(1):45–54. doi: 10.1002/jha2.593 (PMC9928637; doi:10.1002/jha2.593)
Supplement: Supplementary file 1 — Supp Table information [file JHA2-4-45-s001.docx]

**Supplementary Tables**

**TABLE S1. IRRs, AEs and SAEs temporally associated with CT-P10 treatment for the overall cohort**

| **IRR, or AEs associated with CT-P10 treatment in patients with DLBCL** | **Overall (n=389^1^)** |
| --- | --- |
| Proportion of patients experiencing AEs at index or post-index, n (%) |  |
| AEs at index or post-index | 351 (90%) |
| None recorded | 38 (10%) |
| Proportion of patients experiencing non-serious AEs at index or post-index, n (%) |  |
| Non-serious AEs at index or post-index | 324 (83%) |
| None recorded | 65 (17%) |
| Proportion of patients experiencing SAEs at index or post-index, n (% of 387) |  |
| SAEs experienced at index or post-index | 168 (43%) |
| None recorded | 219 (57%) |
| Missing data^2^ | 2 |
| Grade of AEs experienced at index or post-index, n (% of 2,337 events) |  |
| Grade 1 | 1153 (49%) |
| Grade 2 | 715 (31%) |
| Grade 3 | 369 (16%) |
| Grade 4 | 78 (3%) |
| Grade 5 | 22 (1%) |
| Grade not recorded | 167 |
| Relatedness of non-serious AEs experienced at index or post-index, n (% of 2,100 events)^3,*^ |  |
| Definitely related | 27 (1%) |
| Probably related | 44 (2%) |
| Possibly related | 109 (5%) |
| Unlikely to be related | 103 (5%) |
| Not related | 637 (30%) |
| Relationship not recorded | 1,180 (56%) |
| Relatedness of SAEs experienced at index or post-index, n (% of 401 events)^4,*^ |  |
| Definitely related | 13 (3%) |
| Probably related | 5 (1%) |
| Possibly related | 38 (9%) |
| Unlikely to be related | 14 (3%) |
| Not related | 123 (31%) |
| Relationship not recorded | 208 (52%) |
| Proportion of patients experiencing any IRRs on Day 1 or Day 2 post-index event, n (%) |  |
| IRR experienced | 61 (16%) |
| No IRR experienced | 328 (84%) |
| Abbreviations: AE, adverse event; IRR, infusion-related reaction; SAE, serious adverse event.  ^1^Unless otherwise stated.  ^2^A total of 2 patients had AEs with unavailable SAE criteria, with no other SAEs recorded.  ^3^In non-serious AEs with relatedness to CT-P10 recorded (n=920 events), 3% (n=27/920) were definitely related, 5% (n=44/920) were probably related, 12% (n=109/920) were possibly related, 11% (n=103/920) were unlikely to be related and 69% (n=637/920) were considered not related to CT-P10 treatment.  ^4^In SAEs with relatedness to CT-P10 recorded (n=193 events), 7% (n=13/193) were found to be definitely related, 3% (n=5/193) were probably related, 20% (n=38/193) were possibly related, 7% (n=14/193) were unlikely to be related and 64% (n=123/193) were not related to CT-P10 treatment.  *Data are not mutually exclusive per patient. | |

**TABLE S2. IRRs, AEs and SAEs temporally associated with first-line CT-P10 treatment**

| **IRR, or AEs associated with CT-P10 treatment in patients with DLBCL** | **Overall (n=328^1^)** |
| --- | --- |
| Proportion of patients experiencing AEs at index or post-index, n (%) |  |
| AEs at index or post-index | 297 (91%) |
| None recorded | 31 (9%) |
| Proportion of patients experiencing non-serious AEs at index or post-index, n (%) |  |
| Non-serious AEs at index or post-index | 275 (84%) |
| None recorded | 53 (16%) |
| Proportion of patients experiencing SAEs at index or post-index, n (% of 326) |  |
| SAEs experienced at index or post-index | 146 (45%) |
| None recorded | 180 (55%) |
| Missing data^2^ | 2 |
| Grade of AEs experienced at index or post-index, n (% of 1,911 events) |  |
| Grade 1 | 938 (49%) |
| Grade 2 | 584 (31%) |
| Grade 3 | 300 (16%) |
| Grade 4 | 69 (4%) |
| Grade 5 | 20 (1%) |
| Grade not recorded | 146 |
| Relatedness of non-serious AEs experienced at index or post-index, n (% of 1,710 events)^3,*^ |  |
| Definitely related | 19 (1%) |
| Probably related | 41 (2%) |
| Possibly related | 78 (5%) |
| Unlikely to be related | 84 (5%) |
| Not related | 502 (29%) |
| Relationship not recorded | 986 (58%) |
| Relatedness of SAEs experienced at index or post-index, n (% of 345 events)^4,*^ |  |
| Definitely related | 12 (3%) |
| Probably related | 4 (1%) |
| Possibly related | 35 (10%) |
| Unlikely to be related | 12 (3%) |
| Not related | 106 (31%) |
| Relationship not recorded | 176 (51%) |
| Proportion of patients experiencing any IRRs on Day 1 or Day 2 post-index event, n (%) |  |
| IRR experienced | 50 (15%) |
| None recorded | 278 (85%) |
| Abbreviations: AE, adverse event; IRR, infusion-related reaction; SAE, serious adverse event.  ^1^Unless otherwise stated.  ^2^A total of 2 patients had AEs with unavailable SAE criteria, with no other SAEs recorded.  ^3^In non-serious AEs with relatedness to CT-P10 recorded (n=724 events), 3% (n=19/724) were definitely related, 6% (n=41/724) were probably related, 11% (n=78/724) were possibly related, 12% (n=84/724) were unlikely to be related and 69% (n=502/724) were considered not related to CT-P10 treatment.  ^4^In SAEs with relatedness to CT-P10 recorded (n=169 events), 7% (n=12/169) were found to be definitely related, 2% (n=4/169) were probably related, 21% (n=35/169) were possibly related, 7% (n=12/169) were unlikely to be related and 63% (n=106/169) were not related to CT-P10 treatment.  *Data are not mutually exclusive per patient. | |

**TABLE S3 Additional AE data: AEs experienced at index or post-index categorized by system organ class**

| **AEs by System Organ Class (SOC)** | **n (patients)^1^** | **% (n = 389)** | **n (events)** | **% (n=2,504)** |
| --- | --- | --- | --- | --- |
| Blood and lymphatic system disorders | 136 | 35% | 273 | 11% |
| Cardiac disorders | 49 | 13% | 58 | 2% |
| Congenital, familial and genetic disorders | 3 | 1% | 3 | 0% |
| Ear and labyrinth disorders | 10 | 3% | 10 | 0% |
| Endocrine disorders | 5 | 1% | 5 | 0% |
| Eye disorders | 14 | 4% | 16 | 1% |
| Gastrointestinal disorders | 209 | 54% | 511 | 20% |
| General disorders and administration site conditions | 220 | 57% | 409 | 16% |
| Hepatobiliary disorders | 9 | 2% | 9 | 0% |
| Immune system disorders | 11 | 3% | 12 | 0% |
| Infections and infestations | 134 | 34% | 225 | 9% |
| Injury, poisoning and procedural complications | 26 | 7% | 34 | 1% |
| Investigations | 61 | 16% | 161 | 6% |
| Metabolism and nutrition disorders | 45 | 12% | 54 | 2% |
| Muscle disorders | 15 | 4% | 15 | 1% |
| Musculoskeletal and connective tissue disorders | 72 | 19% | 92 | 4% |
| Neoplasms benign, malignant and unspecified (including cysts and polyps) | 15 | 4% | 15 | 1% |
| Nervous system disorders | 161 | 41% | 254 | 10% |
| Product issues | 1 | 0% | 1 | 0% |
| Psychiatric disorders | 25 | 6% | 30 | 1% |
| Renal and urinary disorders | 33 | 8% | 38 | 2% |
| Reproductive system and breast disorders | 10 | 3% | 14 | 1% |
| Respiratory, thoracic and mediastinal disorders | 75 | 19% | 104 | 4% |
| Skin and subcutaneous tissue disorders | 68 | 17% | 95 | 4% |
| Surgical and medical procedures | 10 | 3% | 11 | 0% |
| Vascular disorders | 49 | 13% | 55 | 2% |
| ^1^Denominator for n (patients) is the number of patients with an AE recorded (i.e., all patients).  SOCs are not mutually exclusive per patient. | | | | |

**TABLE S4 Upper and lower 95% confidence interval estimations for percentage survival based on 300 and 500 patients**

| **Original estimates for 500 patients** | | | **Estimates for 300 patients** | | |
| --- | --- | --- | --- | --- | --- |
| **% Survival** | **95% Lower Confidence Limit** | **95% Upper Confidence Limit** | **% Survival** | **95% Lower Confidence Limit** | **95% Upper Confidence Limit** |
| 5% | 3.30% | 7.30% | 5% | 2.80% | 8.10% |
| 10% | 7.50% | 13.00% | 10% | 6.80% | 14.00% |
| 15% | 12.00% | 18.40% | 15% | 11.20% | 19.60% |
| 20% | 16.60% | 23.80% | 20% | 15.60% | 25.00% |
| 25% | 21.30% | 29.00% | 25% | 20.20% | 30.30% |
| 30% | 26.00% | 34.20% | 30% | 24.90% | 35.50% |
| 35% | 30.80% | 39.40% | 35% | 29.60% | 40.70% |
| 40% | 35.70% | 44.40% | 40% | 34.40% | 45.80% |
| 45% | 40.60% | 49.50% | 45% | 39.30% | 50.80% |
| 50% | 45.50% | 54.50% | 50% | 44.20% | 55.80% |
| 55% | 50.50% | 59.40% | 55% | 49.20% | 60.70% |
| 60% | 55.60% | 64.30% | 60% | 54.20% | 65.60% |
| 65% | 60.60% | 69.20% | 65% | 59.30% | 70.40% |
| 70% | 65.80% | 74.00% | 70% | 64.50% | 75.10% |
| 75% | 71.00% | 78.70% | 75% | 69.70% | 79.80% |
| 80% | 76.20% | 83.40% | 80% | 75.00% | 84.40% |
| 85% | 81.60% | 88.00% | 85% | 80.40% | 88.80% |
| 90% | 87.00% | 92.50% | 90% | 86.00% | 93.20% |
| 95% | 92.70% | 96.70% | 95% | 91.90% | 97.20% |
