# Supplementary material for: Real‐world clinical effectiveness and safety of CT‐P10 in patients with diffuse large B‐cell lymphoma: An observational study in Europe
Source: EJHaem. 2022 Nov 6;4(1):45–54. doi: 10.1002/jha2.593 (PMC9928637; doi:10.1002/jha2.593)
Supplement: Supplementary file 2 — Supplementary methods [file JHA2-4-45-s002.docx]

**Supplementary methods**

***Patient consent and local approval***

In relation to country-specific consent processes, in accordance with French regulations, where patient consent is not required for some non-interventional investigations a ‘non-opposition’ model was used, whereby patients or their next of kin were sent information about the study by post and data collection proceeded if they did not raise an opposition within 10 working days; consent was obtained for all living patients from the UK, Spain, Germany and Italy. For UK and Italian centers, deceased patients’ data were collected by the direct care team to preserve confidentiality; no deceased patients were eligible for inclusion from Spanish and German centers. In the UK, approval was granted (reference: 18/LO/0867) from an independent National Health Service Research Ethics Committee (EC) via the Health Research Authority system, followed by study approval from each individual center via its respective research and development departments. In France, the study was considered “hors loi Jardé” and so did not require submission to an ethics committee or regulatory authority (RA). In addition, the study was carried out according to the MR-004 and declared to the Commission Nationale de l'informatique et des libertés (reference: 2209994 v0 dated 22 November 2018). The Direction de la Recherche Clinique et de l'Innovation (hospital administration) was informed regarding the start and end dates of the study.

In Spain, the study was submitted to the RA (Agencia Española del Medicamento y Productos Sanitarios) for classification (classification was confirmed as an EPA-SP study). The resulting classification letter was approved by the central ethics committee (EC; Hospital Morales Meseguer), as per Spanish law.

For Italian centers, approval was granted by the local EC for each center (Bologna center was approved by the local EC [Comitato Etico di Area Vasta Emilia Centro - c/o A.O.U. Policlinico S.Orsola Malpighi], reference prot 588/2018/Oss/AOUBo; Firenze center was approved by the local EC [Comitato Etico Area Vasta Centro - c/o A.O.U. Careggi], reference 13723_oss; Siena center was approved by the local EC [Comitato Etico Area Vasta Sud Est - c/o A.O.U. Senese], reference prot. 13734; Aviano Center was approved by the local EC [Comitato Etico Unico Regionale - c/o Direzione Scientifica del CRO di Aviano] reference, CEUR 2018-OS-105-CRO).

In Germany, the study was submitted to the EC of the Landesärztekammer Hessen (reference FF 123/2018). The study was also submitted by the principal investigators to their local ECs to obtain ethical advice (in this study: Ethikkommission der Ärztekammer Westfalen-Lippe – reference 2019-350-b-S; Ärztekammer Schleswig Holstein – reference 057/19 m; Ethik-Kommission der Ärztekammer Sachsen-Anhalt – reference 42/19). The higher federal authority—in this case, the Paul-Ehrlich-Institut (PEI) – reference NIS449—was notified of the study. Furthermore, the study was reported to the National Association of Statutory Health Insurance Physicians (Kassenärztliche Bundesvereingungen [KBV] – reference A70116, the Central Federal Association of Health Insurance Funds (Spitzenverband Bund der Krankenkassen [GKV] - reference 10742) and the Association of Private Health Insurances (Verband der Privaten Krankenkassen [PKV]).

***Statistical analyses***

The proposed study size of 500 patients was initially proposed to ensure a sufficient sample size to allow for key descriptive analyses to be representative of the sample population as a whole as well as any relevant subgroups, whilst also considering recruitment feasibility ^20^. Based on a previously reported PFS rate over 3 years of approximately 55%^21^ and the target sample size of 500 patients, a 95% CI between 50.5%-59.4% was expected in the present study, which was considered to represent adequate precision to characterise this population. The proportion of patients experiencing a complete or partial response in the above-referenced study^21^ was approximately 75%, which would translate into a 95% CI between 71.0% and 78.7% with a sample size of 500 patients. The actual sample size for this study was n=389, as opposed to the planned n=500 that the initial precision estimates were based upon.

Time-to-event analyses were conducted using the Kaplan-Meier (KM) method and displayed using KM plots from the index date until the date of event or censoring. For OS, patients known to be alive or lost to follow-up were censored at 30-months post-index date or the date of the last recorded hospital visit within 30-months post index, respectively. For PFS, patients were censored on the date of the last documented response assessment in the medical records (this included the Revized Response Criteria for Malignant Lymphoma^22^, if these criteria were used and documented locally). OS and PFS rates (with 95% CI) were calculated at 12-, 18- and 30 months post-index.

For time to complete or partial response, patients were not censored – for patients with ≥1 record of complete response the time from index until the first documentation was used.

OS and PFS for patients where CT-P10 was the first line of treatment were displayed descriptively using Kaplan–Meier (KM) plots from the index date until the date of the event or censoring. OS and PFS rates were calculated at 12-, 18- and 30-months post-index date.

The proportion of patients assessed as having a best response of complete response, partial response, stable disease or progressive disease in the 30 months post-index, were calculated. In addition, the first response recorded that was at least 3 months, and at least 6 months post-index was assessed.

Treatment patterns for CT-P10 were described for the entire study period. Summary statistics were displayed for the treatment dose and treatment duration, and a distribution for the treatment discontinuation reason.

Due to the descriptive nature of this study, no analyses to control for confounding were carried out. In relation to missing data, where dates were ambiguous because of missing days, standard imputation was applied: where day was missing, the 15^th^ of the month was assumed if sensible according to the scale of the measurements. Aside from this, where data were missing from the original medical record, the affected analyses were conducted using only the results of those patients with data available and the number included in each analysis was stated. No other data imputation was carried out. The number of patients with data missing was reported for each study variable.
